# Corresponding anatomical of the macaque superior parietal lobule areas 5 (PE) subdivision reveal similar connectivity patterns with humans

**DOI:** 10.3389/fnins.2022.964310

**Published:** 2022-10-04

**Authors:** Qianshan Wang, Yue Wang, Wenyi Xu, Xiaofeng Chen, Xueqi Li, Qi Li, Haifang Li

**Affiliations:** College of Information and Computer, Taiyuan University of Technology, Taiyuan, China

**Keywords:** brain region subdivision, diffusion tensor imaging, cross-species comparison, brain region homology, superior parietal lobule (SPL)

## Abstract

Using the animal brain as a cross-species tool for human brain research based on imaging features can provide more potential to reveal comprehensive human brain analysis. Previous studies have shown that human Brodmann area 5 (BA5) and macaque PE are homologous regions. They are both involved in processes depth and direction information during the touch process in the arm movement. However, recent studies show that both BA5 and PE are not homogeneous. According to the cytoarchitecture, BA5 is subdivided into three different subregions, and PE can be subdivided into PEl, PEla, and PEm. The species homologous relationship among the subregions is not clear between BA5 and PE. At the same time, the subdivision of PE based on the anatomical connection of white matter fiber bundles needs more verification. This research subdivided the PE of macaques based on the anatomical connection of white matter fiber bundles. Two PE subregions are defined based on probabilistic fiber tracking, one on the anterior side and the other on the dorsal side. Finally, the research draws connectivity fingerprints with predefined homologous target areas for the BA5 and PE subregions to reveal the characteristics of structure and functions and gives the homologous correspondence identified.

## Introduction

The development of brain science depends on the progress of innovative thinking and research methodology, but the living experiments of humans restrict the development of brain science to a certain extent. Cross-species comparison can provide a basis for verifying the assumptions and future research results in the human brain. Due to the similarity and evolutionary homology between the brains of primates, the non-human primate (NHP) brain is a transitional model for human brain research. The macaque brain is a classic transition model, and many invasive experiments can use this model to reflect the working and pathological mechanism of the human brain (Thiebaut de Schotten et al., 2019; Xia et al., 2019a). The accumulation and disclosure of brain image data of humans and non-humans, such as the Human Connectome Project (HCP) (Van Essen et al., 2013) and PRIMatE Data Exchange (PRIME-DE) project (Milham et al., 2018), have laid the foundation for the comparison of cross-species.

Magnetic resonance imaging (MRI) research can support future human brain discovery and hypothesis verification in cross-species comparison. At the same time, it can help identify finer subdivisions of brain regions and reveal their functional and neural circuit characteristics. Functional MRI (fMRI) and diffusion MRI (dMRI) compare functional and structural connections between species. Xia et al. (2019b) determined that the striatal emotion processing network is a conservative region. The homology of cortical projection sources in these regions is determined by the proportion of projections to these conservative regions. Furthermore, some imaging studies are based on anatomical connection patterns, such as exploring comparable characteristics between species through their structural similarity based on homologous brain regions and homologous white matter bundles (Wang et al., 2019). Researchers have also collected functional connectivity characteristics from specific task states of humans and macaques for cross-species comparison (Deco et al., 2013). Through a cross-species comparative study, the researchers found some interesting phenomena. For example, the primary and intermediate visual areas of humans are more introverted than those of macaques (Fattori et al., 2012), and the functional structures of the anterior cuneate nucleus (PCun) of humans and macaques are similar (Wang et al., 2019).

The superior parietal lobule (SPL) is involved in integrating information from visual and somatosensory cortical regions to perform stretching and grasping movements (Passarelli et al., 2021). Most research on macaque SPL focuses on PE, PEc, and V6A. PE is located on the posterior parietal cortex (PPC) and is essentially an advanced somatosensory area dominated by somatosensory input, which participates in the motor processing of depth and direction information in the process of touch (Battaglia-Mayer et al., 2013). The latest research found that PE, PEc and V6A are all involved in the regulation of gaze and arm static position, especially the PE area, which has a more independent coding ability (De Vitis et al., 2022). Pandya and Seltzer (1982) confirmed the existence of PE and defined it as a cytoarchitecture of homogeneous entities. Some studies have shown that 5l and 5m are equivalent to the PE, while 5Ci is equivalent to the PEci of macaques (Scheperjans et al., 2005). A recent neural tracer study shows that PE is not homogeneous. This study found that according to the cytoarchitecture and the average density of some examined receptors and layered distribution patterns, PE can be subdivided into three parts: PEla (lateral-anterior PE), PEl (lateral PE), and PEm (medial PE) (Impieri et al., 2019). However, the functional connectivity pattern and anatomical connectivity pattern of PE subregions have not been studied, and it is not clear whether subdivisions based on cytoarchitecture can match the functional connectivity patterns and anatomical connectivity patterns. At the same time, whether humans and macaques share the connectivity pattern is not clear. Therefore, it is necessary to perform medical imaging histological analysis of the PE subregion.

Currently, connectivity-based parcellation (CBP) has been widely recognized, and its results are consistent with cytoarchitectonic mapping results (Liu et al., 2013; Wang et al., 2016; Ren et al., 2019). The main criterion of brain region segmentation in CBP is the connection information of brain regions. The basic assumption of this method is that each voxel or node belonging to the same brain region has a very similar connection pattern. Behrens used DTI data to segment the thalamus and verified its results (Behrens and Johansen-Berg, 2005). Johansen-Berg used the probability tracking method to track the nerve fibers of the auxiliary motor area and used the cross-correlation method to divide the auxiliary motor area into two regions (Johansen-Berg et al., 2005). Anwander used the same method to resegment the Broca region and the K-means clustering method to obtain three subregions (Anwander et al., 2007). Beckmann applied an iterative K-means clustering method to the segmentation of the cingulate gyrus, and the segmentation results of 9 subregions were obtained (Beckmann et al., 2009). Therefore, the CBP can provide precise structural PE subregion segmentation results.

In this study, the researcher used probabilistic fiber tracking techniques to calculate the anatomical connectivity of the voxels in the PE region and subdivided the PE region into four different subregion schemes. By analyzing the evaluation indexes of these schemes, the subdivision scheme of two regions is selected. Then, the researcher used each subregion’s anatomical and functional connectivity to verify the subdivision result. At the same time, the research team drew 19 predefined homologous target regions for cross-species analysis, and the anatomical connection fingerprints and functional connection fingerprints of BA5 (5L, 5m) and PE (two subregion schemes) with these homologous target regions were calculated. Furthermore, we conducted a cross-species analysis of the subregion area of human BA5 (5L, 5m) and macaque PE. This research can help study the process of arms motivation control and touch in the brain. It is also helpful in analyzing neurosurgical localization and provides a basis for clinical diagnosis and rehabilitation training of human arm movement.

## Materials and methods

### Dataset

#### Human data

This research collected human datasets from the publicly available datasets released by the Human Young Adult projects of Human Connectome Project (HCP) (Van Essen et al., 2013). The human data were acquired using a 3.0 T MR system. This dataset includes 22 healthy subjects (13 males and 9 females, age range = 22–35 years old) and selected their corresponding preprocessed structural MRI (sMRI) and diffusion MRI, where sMRI data include 0.7 mm high-resolution isotropic T1 weight MRI (TR/TE = 2,400/2.14 ms, TI = 1,000 ms, FA = 8°, voxel resolution = 0.7 × 0.7 × 0.7 mm), diffusion MRI data (acquired the spin–echo EPI sequence, slice = 111, TR/TE = 5,520/89.5 ms, FA = 78°, voxel resolution = 1.25 × 1.25 × 1.25 mm, diffusion weighting consisting of 1,000, 2,000, 3,000 s/mm^2^, and 18 non-weighting epochs), and rest-fMRI data (acquired using the gradient-echo EPI sequence, TR/TE = 720/33.1 ms, FA = 52°, voxel resolution = 2 × 2 × 2 mm, scanning matrix = 104 × 90 acquisition time = 14.55 min, and 1,200 frames are collected).

#### Macaque data

This research collected macaque datasets from the publicly available dataset released by the PRIMatE Data Exchange (PRIME-DE) project (Milham et al., 2018). The macaque dataset includes 18 macaques (all female, age distribution: 18.5–22.5 years) from the University of California, Davis, UC-Davis. The data of these macaques were collected using Siemens Skyra 3 T’s 4-channel flip coil scanning. The bred macaques met the UC-Davis IACUC ethics certification. This dataset includes T1 weight MRI (TR/TE = 2,500/3.65 ms, TI = 1,100 ms, FA = 7°, voxel resolution = 0.3 × 0.3 × 0.3 mm), diffusion MRI (TR/TE = 6,400/115 ms, voxel resolution = 1.4 × 1.4 × 1.4 mm, diffusion weighting consisting of 1,600 s/mm^2^ and 800 s/mm^2^), and rest-fMRI (acquired the gradient-echo EPI sequence, TR/TE = 1,600/24 ms, voxel resolution = 1.4 × 1.4 × 1.4 mm). The researcher used these data as the elderly data.

#### Diffusion magnetic resonance imaging data preprocessing

FSL software was used to preprocess the diffusion MRI data, and the preprocessing included the five following steps: (1) Space correction and head movement realign: use the FDT tools in FSL to distortion correction, and the eddy current and head movement of diffusion MRI are corrected (Smith et al., 2004); (2) Brain tissue extraction: our researcher used the ResTLU-net tool designed by our team to extract brain tissue files from brain T1 and diffusion images and remove non-brain tissue (Wang et al., 2022); (3) Individual image space registration: use the FNIRT tool to register the T1-weighted image to the subject’s diffusion image space; (4) Atlases template space registration: register the human and macaque T1 images to the standard MNI152 and D99 templates, respectively. To obtain a more accurate registration effect, the research team adopts a two-step registration method and selects individuals with better registration results to form the experimental dataset. (5) Adopt BEDPOSTX runs Markov chain Monte Carlo stands for modeling crossing Fibers. It creates all the files necessary for running probabilistic tractography.

### Resting-state functional magnetic resonance imaging data preprocessing

The resting-state fMRI data preprocessing process includes the six steps as follows: (1) Time correction (slice timing): the middle layer is selected as the reference, and then the remaining layers are aligned to this layer to eliminate the impact of different acquisition times on the data; (2) Head movement correction (Realign): If there is a translation greater than 2.5 mm in the head movement image, the data with a rotation angle more significant than 2.5° will be discarded; (3) Spatial normalization (Normalization) normalizes the image space after head movement correction to the Montreal Neuropathy Research Institute (MNI) standard head anatomy template and resamples with voxels; (4) Filter: use slow-5 (0.01–0.027 Hz), slow-4 (0.027–0.073 Hz), Slow-3 (0.073–0.198 Hz) and slow-2 (0.198–0.25 Hz) frequencies are filtered to eliminate the influence of physiological noise (such as breathing, heartbeat, etc.) above and below this frequency band. (5) Smooth: Use a Gaussian check with a full-width, half-height (FWHM) of 6 mm to perform spatial smoothing on the image after spatial normalization to reduce random noise in the image and increase the signal-to-noise ratio so that the data are obtained from the space. The resolution is easier to compare. (6) Interference covariate regression: To remove the influence of physiological factors, the white matter, cerebrospinal fluid, head movement, and other covariates were regressed. The above steps are implemented through the DPABI tool (Yan et al., 2016).

### Region of interestregion selection

**FIGURE 1:**
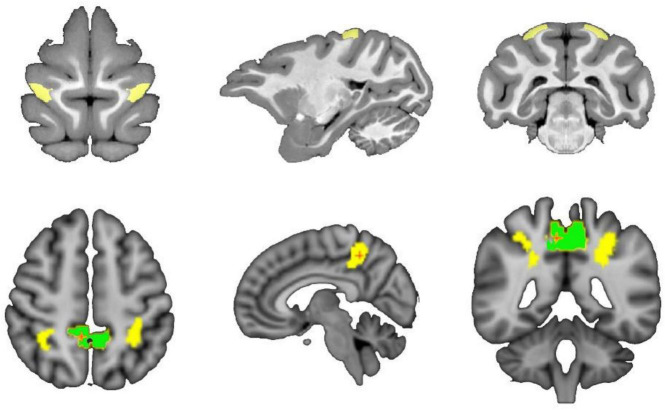
Schematic diagram of the spatial location of PE in macaques and 5L and 5m in humans. The top of the schematic is the macaque brain in the D99 atlas, and the yellow area is the PE area in the macaque brain. The bottom of the schematic diagram is the human brain tissue structure in the MNI standard space, where the yellow area is the 5L area, and the green area is the 5m area.

The researcher obtain the PE area of macaques from D99 atlas v2.0 (Saleem et al., 2021). The maximum probability map (MPM) in individual space is obtained by registration processing, and the PE brain region is extracted. Then, the left and right hemisphere PE regions are manually divided to obtain the seed mask of the bilateral brain region, which is used for subsequent probabilistic fiber tracking and based on connectivity seeds for group analysis. At the same time, the locations of the 5l and 5m regions, which are homologous regions for PE, were labeled under the standard normalized mutual information (NMI) space for comparative analysis across species. The locations of the regions in the D99 and Brainnetome Atlas are shown in Figure 1.

**FIGURE 2:**
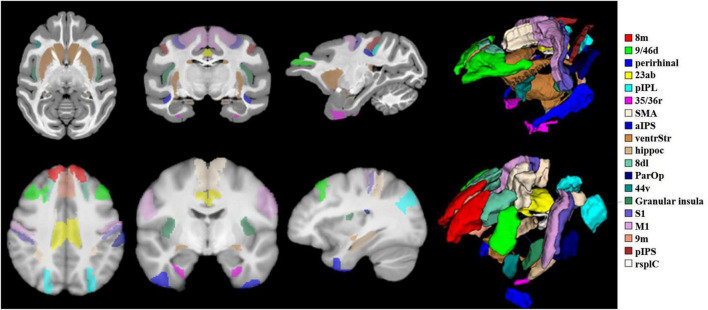
Schematic of 19 target ROIs in the human and macaque brains. The schematic diagram shows the spatial locations of the 19 ROIs selected in this paper under the macaque D99 atlas and the human brainnetome atlas. The relevant regions of interest are marked with 19 different colors, and the relationship between the color and the region is displayed on the far right of the picture. To display the 19 ROIs more intuitively, a 3D stereo image is drawn on the right side of the brain slice.

According to the existing homologous regions of humans and macaques summarized by the research group through the literature, macaques select regions based on the D99 atlas, and humans find the corresponding regions based on the Brainnetome Atlas (Fan et al., 2016). To fully study the functional and structural characteristics of PE regions, 19 different types of homologous brain regions involved in motor, auditory, language, memory, and advanced cognition were selected as follows: 9/46d, 44v, SMA, 8m, M1, S1, ParOp, aIPS, pIPS, pIPL, 23ab, rsplC, perirhinal, ventrStr, hippoc, 35/36r, 9m, 8dL, granular insula. The spatial distribution of the above region of interest (ROI) areas in the standard brain space of humans and macaques is shown in Figure 2.

### Analysis I: Anatomical connectivity-based parcellation of the PE region

#### Probabilistic tractography

Diffusion probabilistic tractography and voxelwise probability distributions were estimated for three directions using the FDT tools (Tomassini et al., 2007). For each voxel in the PE region, 20,000 streamlines were employed, and the connectivity probability between the seed voxel and the remaining voxel was estimated. The resulting anatomical connectivity matrix thus consisted of rows for each PE voxel and columns for each voxel of the whole brain. Next, an asymmetric cross-correlation matrix was generated by multiplying the connectivity matrix by its transposed matrix. The element value is the correlation between the connectivity profile of the seed voxel and the connectivity profile of the seed voxel (Johansen-Berg et al., 2004).

#### Anatomical connectivity-based parcellation of the PE region

Spectral clustering is used to process the similarity matrix to determine 2–5 different numbers of subregional clusters (Wang et al., 2012, 2015; Fan et al., 2014; Xu et al., 2015; Yang et al., 2016). Next, the individual subdivision results of a specific object are transformed from diffusion to MNI space, and a MPM is created for each solution of all objects. The MPM uses the subdivision results of all subjects in the MNI space and is calculated by assigning each voxel in the reference space to the most likely location area. If two regions show the same probability on a particular voxel, then this voxel is assigned to the region with a higher average probability of 26 voxels (Eickhoff et al., 2005; Li et al., 2017; He et al., 2020).

#### Determining the optimal number of subregions of the PE

The Dice coefficient is used to evaluate the degree of overlap between grouping results based on anatomical connectivity to identify the corresponding structure topology of PE, thereby determining the best grouping of PE neutron regions. A higher Dice coefficient is considered to reflect better PE segmentation. The average Crame’s V (CV) is also used to measure the spatial distribution consistency of the regions between individuals. The researcher randomly divides the samples into two groups, the CV values of the results of the two groups are calculated, and the process is repeated 1,000 times to calculate the average CV value. The better partitioning scheme with a CV value closer to 1. NMI, the stable partitioning scheme, is determined with a higher MNI value. The loss or gain of information between two partitioning schemes is quantified using the variation of information (VI) indicator, which can be used to determine the most stable partitioning scheme by a lower value. Finally, the research team performed median filtering on the clustering results and matched the PE subregions to the D99 template.

### Analysis II: Anatomical connectivity patterns cross-species comparisons of PE

#### Anatomical connectivity mapping

Probabilistic tractography was performed using FSL for 5,000 streamlines per voxel from the target in human brain research. Considering that the imaging quality and voxel resolution of macaque brain diffusion MRI are lower than those of the human brain, to obtain accurate anatomical connectivity information of the macaque, probabilistic tractography was performed using 50,000 streamlines per voxel from the macaque PE region and its subregions to the target ROI mapping the anatomical connections between each voxel. Finally, the probability of anatomical connectivity from each region to each target brain region was determined.

#### Comparisons of anatomical connectivity patterns

To further evaluate the connectivity fingerprints between humans and macaques, the connectivity values for each region were normalized using Equation 1. The maximum connectivity probability between the PE subregions and any target brain region was 1, while the minimum connectivity probability was 0.


(1) $$P_{i,j}^{{}^{\prime }}=\frac{p_{i,j}-m\,a\,x\,\left(p_{i}\right)}{m\,a\,x\,\left(p_{i}\right)-m\,i\,n\,\left(p_{i}\right)}$$

where *P*_*i, j*_ is the connectivity probability between the *i* PE subregion and the *j* target brain region. *Max*(*p*_*i*_) is the maximum connectivity probability between the PE subregion and all target brain regions, while *min*(*p*_*i*_) is the minimum connectivity probability between the PE subregion and all target brain regions. The normalized anatomical connectivity probability values were used to construct a connectivity fingerprint for each PE subregion. Finally, the cosine similarity and Manhattan distance between human and macaque fingerprints were calculated to characterize the similarity between humans and macaques.


(2) $$C\,S=\frac{\sum_{i=1}^{n}p_{i}\,\text{×}\,q_{i}}{\sqrt{\sum_{i=1}^{n}p_{i}^{2}}\,\text{×}\,\sqrt{\sum_{i=1}^{n}q_{i}^{2}}}$$

The cosine similarity (CS) is calculated as Equation 2. In Equation 2, *p*,*q* represents two fingerprints to be compared; *n* represents the number of target regions in the fingerprint; and *p*_*i*_, *q*_*i*_ represents the connection value of the *i* target region in the *p*, *q* fingerprints. The value of the cosine similarity calculation ranges from –1 to 1: the closer the value is to 1, the more similar the connectivity of the two brain regions is, that is, the greater the likelihood that the two brain regions are homologous; the closer the value is to 0, which indicates that the two brain regions are independent of each other, and the less likely it is to be homologous; the value is close to –1, indicating that the two brain regions are negatively correlated.


(3) $$M\,D=\sum_{i=1}^{n}\left|p_{i}-q_{i}\right|$$

The Manhattan Distance (MD) is calculated using Equation 3. In Equation 3, *p* and *q* are the two vectors of the connected fingerprint, and *i* represents the target regions of the fingerprint. The smaller the value of the Manhattan distance, the higher the similarity of the two vectors, and the more similar the connectivity of the two brain regions, that is, the greater the possibility that the two brain regions are homologous.

### Analysis III: Functional connectivity patterns cross-species comparisons of PE

#### Delineating the subregion-specific functional networks

The research team used the DPABI tool to perform the resting-state functional connection of 197 brain regions corresponding to PE based on the D99 template (Yan et al., 2016). Then, the Pearson correlation coefficients between PE and them were calculated. In statistics, the Pearson product-moment correlation coefficient (Pearson’s r) is used to measure the correlation between two variables, X and Y (linear correlation), and its value is between –1 and 1. It is a widely used indicator to measure the degree of correlation between brain regions in resting-state function data. The research team used the Pearson correlation coefficient as the eigenvalue to construct the functional connectivity network between PE (subregions) and the predefined ROIs.

#### Comparison of functional connectivity patterns

First, the research team mapped the functional connectivity network of the PE (and the PE subregions divided by the team) with predefined ROIs to determine the connection patterns of different brain regions. The team then calculated the functional connectivity patterns of the human brain’s 5L and 5m regions. Then, the cosine similarity and Manhattan distance between different subregions are calculated to find the most similar subregion matching between the human brain and macaque brain.

## Results

### Analysis I: Anatomical connectivity-based parcellation of the PE region

#### Cluster partitioning of the area PE

**FIGURE 3:**
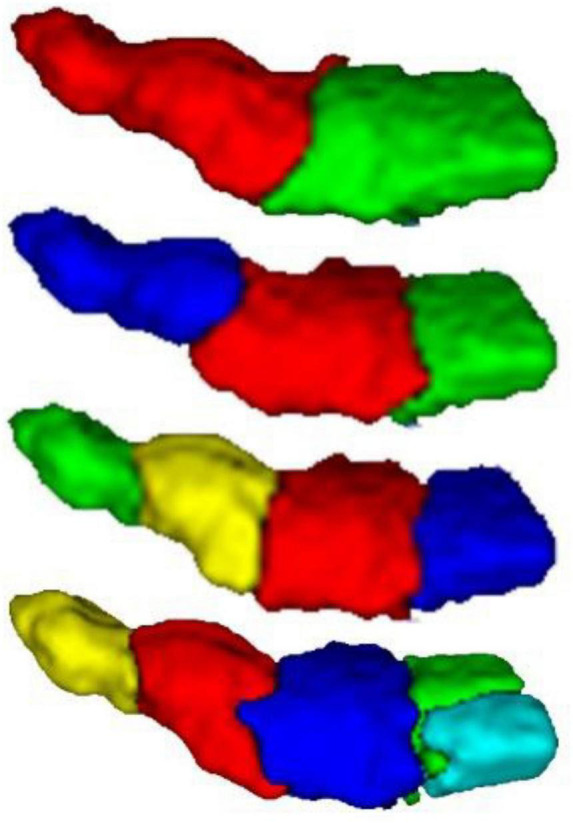
3D result diagram of different PE subregion schemes. From top to bottom, there are clustering results for brains with 2, 3, 4, and 5.

The MPM of PE clustered into 2, 3, 4, and 5 subregions was obtained from the grouping based on anatomical connectivity. Figure 3 is a schematic diagram of the spatial location distribution of the four regional clustering results. Each subregion of the brain image is divided into different clusters by color, which is divided into 2, 3, 4, and 5 subregions from top to bottom.

To determine the optimal number of subregions of the PE, the evaluation indexes of four subdivision schemes of the left and right hemispheres are calculated (Dice, NMI, CV, and VI), and the results are shown in Table 1. The results showed that Dice, CV and NMI were the optimal outcomes in the subdivided subregion results when the clustering num was set to 2, as in the left brain subregion results where the CV value was 0.521, indicating high consistency of regional distribution among individuals. The Dice coefficient was 0.416, indicating a better topology for this grouping. The NMI parameter value was 0.546, indicating that the grouping results of this scheme exhibited superior stability on different individuals compared with other grouping schemes. Meanwhile, the VI parameter of the bipartition scheme is the smallest at 0.002, indicating that this segmentation scheme loses the least amount of information.

**TABLE 1:** Evaluation indexes of PE segmentation of the left and right brains of macaques.

Pairwise	Hemispheres	Cluster_num			
		2	3	4	5
CV	Left	0.521	0.474	0.438	0.418
Dice		0.416	0.360	0.290	0.234
NMI		0.546	0.536	0.529	0.526
VI		0.002	0.003	0.003	0.003
CV	Right	0.424	0.399	0.386	0.372
Dice		0.328	0.282	0.264	0.231
NMI		0.421	0.418	0.416	0.405
VI		0.0012	0.0013	0.0013	0.0014

**FIGURE 4:**
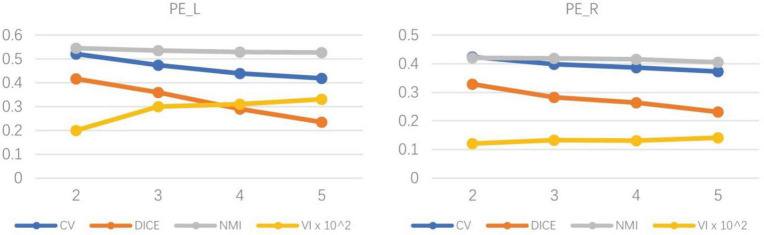
The trend chart of indexes of the left and right hemispheres under different subdivision scheme results. The trend graph on the left shows the evaluation indexes of the four segmentation results of the left hemisphere of the brain, and the trend graph on the right shows the evaluation indexes of the four segmentation results of the right hemisphere of the brain. With the increase in the number of partitions, the evaluation indicators under different partition schemes all decrease to varying degrees.

As shown in Figure 4, the left and right hemispheres of the evaluation indicators showed a consistent trend. Therefore, the researchers believe that dividing the PE brain regions of the left and right hemispheres into two subdivision schemes is the best grouping result. Subsequently, cosine similarity and Manhattan distance between fingerprints of subdivided regions of the left and right hemispheres were calculated, as shown in Table 2. Identify the similar subdivided result within the left and right hemispheres into the same subdivision, which was tentatively named PE_1 (consist with PE_L1 and PE_R1) and PE_ 2 (consist with PE_L2 and PE_R2). In the D99 template space, the spatial location of the space centroid for each subregion is as follows: PE_L1 (95,128,201), PE_L2 (286,134,201), PE_R1 (179,134,201), PE_R2 (187,126,201).

**TABLE 2:** Evaluation of similarity between fingerprints of PE subregion.

Subregion	CS	MD
PE_L1- PE_R1	0.956	0.738
PE_L1- PE_R2	0.940	0.837
PE_L2- PE_R1	0.936	0.878
PE_L2- PE_R2	0.975	0.463

#### Cluster partitioning of the area PE

**FIGURE 5:**
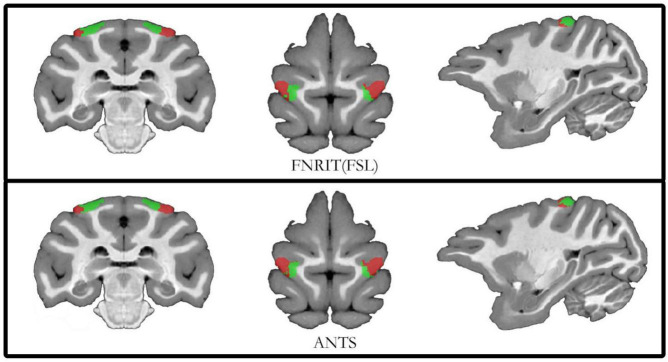
Diagram of subdivision results based on FNIRT and ANTs registration. The top of the schematic shows the results of registration using the FNIRT method. Below the schematic diagram is the result of the registration of the partitions using the ANTs tool.

To ensure the accuracy of the segmentation results, researchers used two image registration tools, FNIRT (FSL) and ANTs, to process the raw data of macaque brain MRI and performed probabilistic tractography calculations and cluster analysis to compare the two segmentation results. The median filtered the clustering results of the two alignment methods and assigned them back to the D99 template with good overlap and the D99 atlas for subsequent subregion correlation experiments (Figure 5). For the partition results obtained by the two registration methods, the Dice, sensitivity, specificity, and intersection over union (IoU) were calculated. The obtained results showed that the effect of registration on the template was better (Table 3). The experimental results show that the Dice, sensitivity, and specificity are all greater than 70%, and the IoU is greater than 0.5. This shows that the results of the PE subregion partition obtained by the two registration methods have a higher coincidence degree, and the experiment can be reproduced.

**TABLE 3:** The spatial overlap effect of the macaque PE subdivision based on FNIRT and ANTs.

Macaque cerebral hemisphere	Dice	Sen.	Spc.	IoU
Left	0.7800	0.8678	0.9999	0.6393
Right	0.7127	0.7943	0.9999	0.5536

### Analysis II: Cross-species comparisons of anatomical connectivity patterns

#### Cluster partitioning of the area PE

**FIGURE 6:**
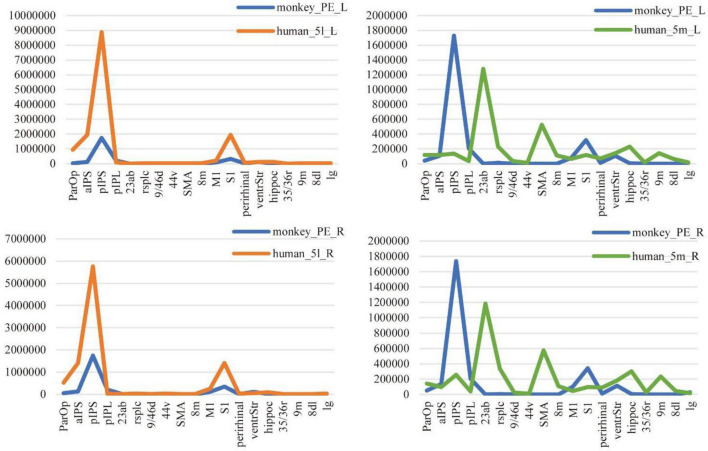
Comparison of anatomical connections between 5L and 5m of humans and the PE of macaques. The trend graph and the X-axis are the 19 ROIs selected in the study, and the Y-axis is the connection frequency from the seed area to the target area calculated in the probabilistic fiber tracking. (Here, the probabilistic fiber tracing of both human and macaque brains is set to 20,000 times per voxel point) The upper left corner image shows the connection of the PE region and 5L region to 19 ROIs, in the left hemisphere of the macaque and human brain. The lower left corner image shows the connection of the PE region and 5L region to 19 ROIs, in the right hemisphere of the macaque and human brain. The upper right corner image shows the connection of the PE region and 5m region to 19 ROIs, in the left hemisphere of the macaque and human brain. The lower right corner image shows the connection of the PE region and 5m region to 19 ROIs, in the right hemisphere of the macaque and human brain.

The comparison method based on connectivity fingerprints brings the brains of the two species into the common coordinate system constructed by the homologous brain regions. The team drew the trend map for the bilateral 5L (5l_L and 5l_R) and 5m (5m_L and 5m_R) regions of the human brain and bilateral PE (PE_L and PE_R) region based on 19 target ROI, shown as shown Figure 6, and calculated the cosine similarity and Manhattan distance between six regions. From Table 4, it can be found that the structural connection pattern of the PE region is closer to human 5L. According to the trend graph, macaque PE and human 51 have a strong trend consistency, and both PE and 51 showed strong connectivity to pIPS and S1. The location of pIPS in the human brain is also located in SPL, and the corresponding region of pIPS in the macaque brain is 5_(PEa), 5_(PEa) itself is adjacent to PE; S1 is the main somatosensory cortex area of humans and macaques, and it is explainable that there is a stable connection (Galletti and Fattori, 2018; Passarelli et al., 2021). According to the above results, the researcher judged that human 5L and macaque PE have a homologous relationship.

**TABLE 4:** Indication of CS and MD in anatomical connectivity fingerprints between human and macaque.

Human-macaque	CS	MD
5l- PE	0.978	0.487
5m- PE	0.166	3.409
5l_L–PE_L	0.978	0.477
5l_R- PE_R	0.977	0.504
5m_L- PE_L	0.114	3.252
5m_R- PE_R	0.198	3.590
5l_L-PE_L1	0.637	2.138
5l_L-PE_L2	0.609	2.071
5m_L-PE_L1	0.128	4.267
5m_L-PE_L2	0.131	3.874
5l_R-PE_R1	0.451	2.435
5l_R-PE_R2	0.379	2.827
5m_R-PE_R1	0.135	3.970
5m_R-PE_R2	0.123	4.261

#### Anatomical connectivity of the PE subregions

**FIGURE 7:**
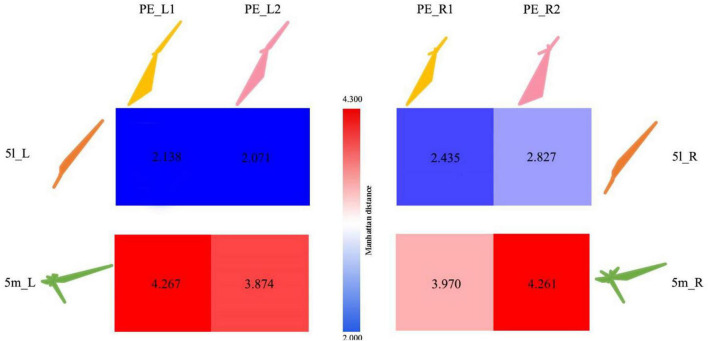
Comparison of anatomical connections between 5L and 5m in humans and subregions of the PE. 5l_L represents the 5L area in the left hemisphere of the human brain; 5l_R represents the 5L area in the right hemisphere of the human brain; 5m_L represents the 5m area in the left hemisphere of the human brain; 5m_R represents the 5m area in the right hemisphere of the human brain area. PE_L1 represents the PE_1 region in the left hemisphere of the macaque brain; PE_L2 represents the PE_2 region in the left hemisphere of the macaque brain; PE_R1 represents the PE_1 region in the right hemisphere of the macaque brain; PE_R2 represents the PE_2 region in the right hemisphere of the macaque brain. The numbers in the squares represent the Manhattan distance between the corresponding areas of the monkey brain and the human brain, with red representing far distance and blue representing near distance.

The Manhattan distance of the connection fingerprints of the bilateral 5L (5l_L and 5l_R) and 5m (5m_L and 5m_R) regions with the PE subregions in Analysis 1 (two-subregion scheme, including PE_L1, PE_L2, PE_R1, PE_R2) is shown in Figure 7. Blue represents a smaller distance from Manhattan, and red indicates a greater difference between the two regions. It can be found that the PE_1 of both hemispheres is similar to human 5L, and it is believed that there is a homologous relationship for PE_1 and 5L. As shown in Table 4, the 5m subregion and PE in this experiment were the same as the 5m as a whole and did not show an obvious homologous relationship with PE, nor did the left and right brains show an obvious corresponding relationship with the subregion. In addition, the PE subregion also shows strong connections to M1, which is the primary motor cortex in humans and macaques (Galletti et al., 2003).

### Analysis III: Functional connectivity patterns cross-species comparisons of PE

#### Resting-state functional connectivity of the PE area

**FIGURE 8:**
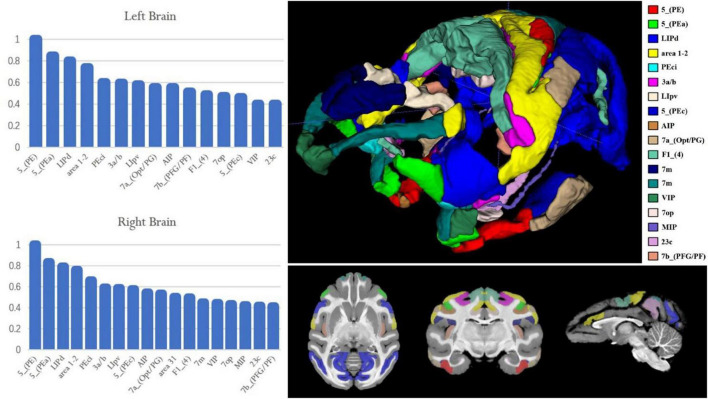
Resting-state functional connectivity of the macaque PE area. The X-axis represents the region of interest used to calculate the connection; the Y-axis represents the value of the Pearson correlation coefficient. The upper left histogram represents the Pearson correlation coefficient between the PE region in the left hemisphere and the region of interest. The lower left histogram represents the Pearson correlation coefficient between the PE region in the right hemisphere and the region of interest. The image on the right shows the ROI region and its spatial location used on the left histogram.

The team performed experiments on the resting-state functional connectivity between brain regions and brain regions according to the division of brain regions in D99 and calculated the Pearson correlation coefficients between PE regions and the rest of the brain regions in the bilateral brains. The researcher selected brain regions with Pearson correlation coefficients higher than 0.4 to draw the percentage bar chart for bilateral PE (Figure 8). It was found that both the right and left brains of macaque monkeys were found to be different in 5_(PEa), LIPd (lateral intraparietal area, dorsal subdivision), 1–2 (somatosensory areas 1 and 2), Peci, 3a/b (somatosensory areas 3a and 3b), and LIpv (lateral intraparietal area, ventral subdivision) were strongly correlated, and the correlations were decreasing in order. The results showed that some brain areas with a weaker correlation with PE function showed some differences between the left and right brains; for example, the correlation between PE and AIP was stronger in the left brain than in 7a_(Opt/PG) and the opposite in the right brain.

**FIGURE 9:**
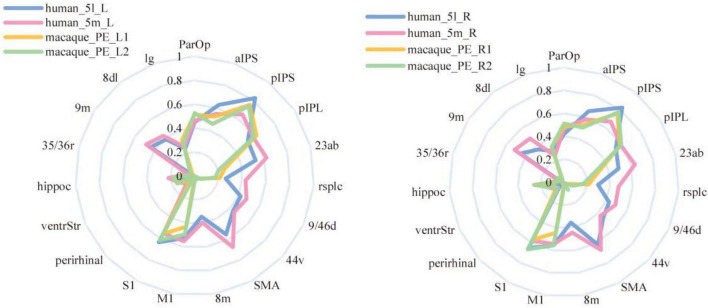
Resting-state functional connectivity fingerprints of PE, 5L, and 5m. The left-hand fingerprint image shows the connections between human and macaque brain regions in the left hemisphere. The right fingerprint image shows connections between human and macaque brain regions associated with the right hemisphere.

The researcher also calculated the CS and MD for the fingerprints of the corresponding brain regions in the bilateral PE region to observe the differences between the left and right PE regions. From Table 5, both 5L and 5m have a strong correlation with PE, 5L is relatively closer to PE, and the overall correlation of the left brain is stronger. Compared with the results of the structural connection, the 5m region shows a higher similarity with the PE region. The functional connection fingerprints of the PE, 5L and 5m regions are shown in Figure 9. The human 5L region, 5m area and macaques PE region also show strong connections in the resting state functional connections to pIPS, S1 and other regions that have previously been prominent in structural connections. At the same time, it also shows new connection characteristics relative to structural connections in other brain regions. The connection of 5L and 5m regions in humans is stronger than that in macaques in 9m, 8 dL, 9/46d, 44v, SMA, 23ab.

**TABLE 5:** Indication of CS and MD in resting-state functional connectivity fingerprints between human and macaque.

Human-macaque	CS	MD
5l–PE	0.859	3.833
5m – PE	0.809	5.713
5l_L-PE_L	0.849	4.287
5l_R-PE_R	0.849	4.007
5m_L-PE_L	0.786	6.294
5m_R-PE_R	0.789	6.682
5l_L-PE_L1	0.841	4.134
5l_L-PE_L2	0.844	4.214
5m_L-PE_L1	0.792	5.598
5m_L-PE_L2	0.794	5.588
5l_R-PE_R1	0.844	4.090
5l_R-PE_R2	0.852	4.146
5m_R-PE_R1	0.795	6.134
5m_R-PE_R2	0.798	5.871

#### Resting-state functional connectivity of the PE subregions

**FIGURE 10:**
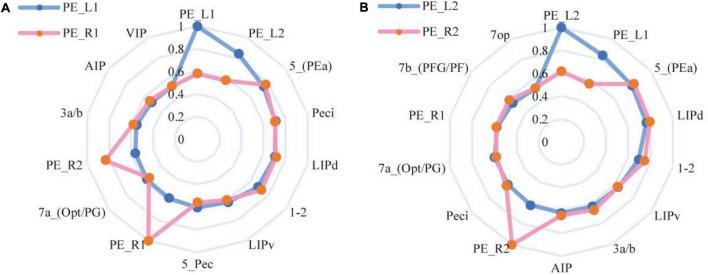
Resting-state functional connectivity fingerprinting of PE subregions. **(A)** The fingerprint shows the related brain regions connections with bilateral hemispheres of the macaque brain PE_1 area. **(B)** The fingerprint shows the related brain regions connections with bilateral hemispheres of the macaque brain PE_2 area.

To investigate the functional connectivity characteristics of PE subregions, the team calculated the Pearson correlation coefficients of bilateral PE subregions with the whole brain. Figure 10A shows the whole-brain resting-state functional connectivity fingerprints of the PE subregions bilaterally, and the regions with Pearson correlation coefficients greater than 0.5 were selected as connectivity points. Figure 10A shows the left hemisphere connectivity fingerprints, and the connectivity target regions were selected starting from the PE left subregion and arranged clockwise in descending order of connectivity strength, while Figure 10B shows the right hemisphere connectivity fingerprints, and the target regions were selected in the same way as the left hemisphere. In the functional correlation analysis, Pearson correlation coefficients shown in the PE subregions of the macaque brain are all the highest correlated with the other area on the same side, followed by the other side of the brain and its corresponding location. For example, for PE_L1: PE_L2 > PE_R1 > PE_R2.

Researchers synthesized the results of the two registration methods, excluding the results with large differences in correlation coefficients, to ensure the reliability of the calculation results. The Dice coefficients based on the two registration methods are PE_L1: [0.968], PE_L2: [0.966], PE_R1: [0.967], and PE_R2: [0.964], which further verifies that the two-subregion schemes of the PE region are valid under the two registration methods.

The CS and MD indicators are given in Table 5. Considering Figure 9, it can be found that 5L seems to have more substantial functional homology with PE_L1, 5m has stronger functional homology with PE_L2, and the Manhattan distance is also closer, which is consistent with the DTI results. Compared with the entire PE region, the similarity between the PE subregion and the 5m subregion is higher than the structural connection. The functional connection similarity between the PE and 5L is higher than 5m, but it is not significant in the PE subregion. However, the results of the Manhattan distance show characteristics consistent with the structural connection. Therefore, the researcher inferred that there is a clear homologous relationship between PE_1 and 5L, and there is a functional homologous relationship between PE_2 and 5m.

## Discussion

### Cellular architecture analysis

In previous studies, differences in the mean density (average density over all layers) and lamellar distribution pattern according to the cellular architecture as well as some examined receptors led to the subdivision of the cellular architecture region PE into three parts (Impieri et al., 2019): the PEla, PEl, and PEm. The PEm and PEla had clearly visible columnar organization, while the PEl was not. In terms of layer thickness, all three regions have similarly well-developed layer III; in contrast, layer IV differs differently: the PEm shows a thicker granular layer, and in the PEla and PEl, the layers became thinner. Another difference is that the border of the white matter is clearly different only in the PEla region. In terms of cell quantity, the PEm area shows a better-stained cytosol with a few large cone cells in layer V, whereas the PEla area shows a clear strip of large cones in layer III, especially in sublayer IIIc. The PEl area shows a strip filled with cone neurons corresponding to layer III. In addition, the tissue particles in PEm were larger, wider, and coarser, whereas the tissue in PEl was smaller, finer, and more compact, with PEla being between them. Therefore, the research speculates that there may be an apparent cooperative junction area in PE.

In this study, a non-invasive method was used to subdivide PE regions using probabilistic fiber tracking based on DTI data and combined with a clustering algorithm, which has been widely used in the field of brain region subdivision, such as fine delineation of the nucleus ambiguous, precuneus, and medial thalamic nucleus (Wang et al., 2019; Xia et al., 2019b; Li et al., 2022). This research determined a convergent PE topographic tissue based on the anatomical connection through white matter fiber bundle tracking clustering, including an anterior PE subregion (PE_1) and a dorsal PE subregion (PE_2). Based on the present results, it is unclear whether to form a complete regional correspondence between the subregions delineated in this research and previous studies. Furthermore, the PE subregions delineated in this study, their respective structural and functional characteristics, and their homologous relationships with human brain regions will be further discussed.

### Anatomical architecture analysis

Before the homology analysis of cross-species structure, the research compares the similarities between bilateral PE_1 and PE_2. There is a very high similarity between the subregions. Differences were shown in the structural connectivity of PE subregions to target regions: the PE_L1 and PE_R1 regions were found to be more connected to the pIPS, which corresponds in the macaque brain to region 5_(PEa), adjacent to the PE region, and has somatic and visual perception functions. The PE_L2 region is more connected to the PE_R2 and ventrStr regions. There is evidence that area ventrStr integrates inputs from the lateral amygdala, hippocampus, prefrontal cortex, and other areas to produce excitatory signals acting on the motor system (outcome prediction) and is able to integrate inputs from the lateral nucleus of the amygdala, hippocampus, prefrontal cortex, and other areas to stimulate or deinhibit goal-directed behavior and integrate declarative memory and procedural memory components that constitute the third memory system (Pennartz et al., 2011).

This study found that the PE, PE subregion, and area 5L showed conspicuous connectivity to the pIPS area and S1 area. Region S1 is the primary somatosensory cortical area in humans and macaques (Dum and Strick, 2002; Grol et al., 2007). According to previous studies, human Brodman area 5 (BA5) is located in the lateral and medial portions of the superior parietal cortex, including the medial part of the intraparietal sulcus that overlaps with areas PE, PEa, and PEc (Saleem and Logothetis, 2012). BA5 is characterized by a dense concentration of SMI-32-positive pyramidal neurons in layers III and V. Recent studies in macaques have also revealed that the PE area in macaques is a somatosensory motor area, which may indicate that the macaque PE region plays a similar role to human BA5. In interneurons, extension direction and depth are mainly encoded by different neuronal populations. Direction signals are more prominent before movement onset, whereas depth processing occurs mainly during and after movement execution. It was shown that the cerebral cortex is strongly involved in the motor processing of depth and direction information during the arrival process. This highlights a trend in the intermediate PPC areas, from the joint encoding of depth and direction signals in the tail of area V6A to the mostly separate processing of the two signals in the tail of area PE (De Vitis et al., 2019).

The anatomical connection architecture shows that both PE subregions may be homologous to humans, and the PE_1 region may be more similar to the human 5L region. In the structural connection, both PE subregions do not show a similar connection mode of the 5m region. Through the similarity of the results, it is found that there is a specific relationship between the strength of structural connectivity reflected by this method and the distance between regions, so the similarity or homologous relationship reflected by structural connection has some limitations. Therefore, the inference of homology with the 5m region mentioned by predecessors needs to be further verified, and functional connectivity is needed to make a more comprehensive discussion.

### Functional architecture analysis

By comparing the results of the resting-state fMRI data calculated in Analysis III, this study found that there is an apparent functional correlation between the PE subregion and the human regions 5L and 5m, and the connection characteristics of the 5L region are more similar to those of PE_1. Region 5m also showed higher similarity with PE_2 in functional connectivity.

Some previous experiments on the monkey PE area were combined to understand the main functional features. Ferraina et al. (2009) found that gaze signals had relatively little effect on PE neural activity during the reach phase. Gaze position modulation in PE may arise from the central median nucleus of the thalamus, which exhibits various eye movement-related signals, even though the association with PE is fragile (Kunimatsu and Tanaka, 2012; Impieri et al., 2018). A study established a sensory mapping experiment. Most medial PE recording points showed somatosensory sensitivity, and many cells responded to stimuli in both upper and lower limbs, suggesting that it plays a role in arm-leg coordination postural control (De Vitis et al., 2019). This is probably because the middle part of the PE in this study is closer to the middle than that studied by Padberg et al. (2019). Most of the PE neurons in this study showed a greater preference for distant space during motor execution and goal-keeping, showing that arm and leg coordination may be more critical for postural adjustment when monkeys reach and hold the furthest target. In medial PE, depth and orientation signals of reach are partially processed by two distinct neuronal subpopulations integrating somatic input from upper and lower extremities.

The present study verified that PE areas have motor control from a functional connectivity perspective and inferred that PE_1 is more oriented toward spatial arm movements, and PE_2 may tend to engage in visuomotor behavior based on the strong and weak relationship to target area connectivity. Monkeys and humans have similar processing in reaching direction and depth. SPL is involved in sensory-motor integration in the primate brain, and reach-related signals have been found in several human SPL subregions, including human V6A, PE and PEci (Cavina-Pratesi et al., 2010; Tosoni et al., 2015; Pitzalis et al., 2019). Martin et al. (2015) indicated that the PPC is activated during reaching peripheral targets. This direction-selective signal is present in areas V6A and 7A and the medial and posterior IPS. Therefore, this research hypothesizes that PE_1 is homologous to human area 5L and that PE_2 is functionally similar to human 5m.

According to the description of the function of the brain region in the brain atlas provided by the Chinese Academy of Sciences (Fan et al., 2016), the 5L_L area of the human brain is sensitive to tasks such as observation and gaze, and it has the tactile recognition of complex patterns and the spatial organization function of movement patterns. The 5lR region is responsible for controlling random movement and imaginative cognition in the flanker inhibition control experiment. Human region 5m achieved cognition of memory and space in completing the n-back task. Therefore, it is hypothesized that the anterior subregion of PE has the function of spatial perception and motor control, and the dorsal subregion of PE has the ability of memory and spatial cognition. The connection-based study of the brain’s PE regions helps construct a cross-species comparison framework, and further investigation can be made into the information processing mechanisms and functions between the human and macaque.

Previously, the segmentation scheme that researchers divided the PE area into 3 subregions based on receptor density was very valuable (Impieri et al., 2019). Therefore, this study chose the subdivision scheme of two subregions as the best, mainly based on connection-based brain tissue segmentation. method evaluation index (Wang et al., 2018). The relationship between our subdivision scheme in two subregions and that in three subregions remains to be further analyzed. From the preliminary observation of the current morphological structure, it can be found that the main parts of PE1 and PE1 together constitute the PE_1 region in our scheme, and a small part of PE1 and PE1 combine with PEm to constitute the PE_2 region in our scheme.

In addition, FSL and ANTs were used for alignment in this study; both methods are commonly used methods in image alignment, and good alignment results were obtained. The partitioning results obtained by the two alignment methods have a high degree of overlap, so the standard coordinates are chosen to locate the subregions in this study. The cross-species comparative analysis further demonstrated that the performance of the subregions obtained by the two methods remained consistent, but there were also differences. Therefore, this research combines the two results and adopts consistent phenomena to draw more reliable conclusions. Finally, the subdivided PE subregions were registered to the macaque D99 brain atlas, and this work laid the foundation for the subsequent studies in this research and provided ideas for refining and supplementing the brain atlas, which will be helpful in future related studies about the brain atlas.

## Conclusion

This study distinguished the anatomical connectivity pattern of each voxel in the PE area, and a new subregion subdivision scheme was obtained by clustering. Then, the partition scheme of the two subregions is determined as the optimal result. To further support the two subdivision schemes of the PE region, the anatomical and functional connectivity patterns were analyzed. Since anatomical connectivity can reflect the functional structure to some extent, resting-state fMRI was used to analyze the functional connectivity of subregions. Finally, the research team decided that the PE could be divided into two subregions, and according to its spatial location, it was determined as the anterior side of PE (PE_1), and the other was located on the dorsal side of PE (PE_2).

In addition, a cross-species comparison between humans and macaques was conducted to explore the homology relationship between this region in the two species. Furthermore, it was found that the anterior side of PE (PE_1) had more similar connectivity features with 5L in the human brain, and 5m showed higher similarity with the dorsal side of PE (PE_2) in functional connectivity as distinct from structural connectivity. In summary, this study identified functional PE topologies and structural connectivity patterns shared by humans and macaques, suggesting that PE has evolved similar roles between species. These findings provide more detailed information on the functional organization of PE and may facilitate future clinical, cognitive, and evolutionary studies in this field. In particular, research on improving the brain-computer interface and the control of bionic prosthetic limbs can provide more precise brain localization.

## Data availability statement

In this research, all datasets were collected from publicly available datasets. The human data are accessed at https://www.humanconnectome.org/study/hcp-young-adult. The macaque data are processed at http://fcon_1000.projects.nitrc.org/indi/PRIMEdownloads.html.

## Ethics statement

The studies involving human participants were reviewed and approved by special member of the Ethics Committee of the academic Committee of Taiyuan University of Technology. The patients/participants provided their written informed consent to participate in this study. The animal study was reviewed and approved by UC-Davis IACUC.

## Author contributions

HL evaluated and guided the experimental design of this research. QW conceived and designed the experiments. WX, XC, and XL collected the data. QW and YW analyzed the results and wrote the manuscript. QL revised the manuscript language. All authors reviewed and approved the manuscript for submission.
